# Intradural Extramedullary Pyogenic Abscess: Incidence, Management, and Clinical Outcomes in 45 Patients With a Mean Follow Up of 2 Years

**DOI:** 10.1177/21925682231151640

**Published:** 2023-01-09

**Authors:** Pavlina Lenga, Stepan Fedorko, Gelo Gülec, cand med, Karl Kiening, Andreas W. Unterberg, Basem Ishak

**Affiliations:** 1 Department of Neurosurgery, 9144 Heidelberg University Hospital, Heidelberg, Germany

**Keywords:** intradural abscess, spinal infection, decompression, comorbidities

## Abstract

**Study Design:**

Retrospective review

**Objectives:**

Spinal intradural extramedullary abscess (SIEA) is a rare disease with an unknown incidence. In this study, we systematically described the clinical course of SIEA in a large cohort with acute onset of neurological illness, assessed the morbidity and mortality rates, and determined the potential risk factors for mortality.

**Methods:**

Electronic medical records of patients diagnosed with SIEA at a single institution for the period between September 2005 and December 2020 were retrieved.

**Results:**

Over a period of 15 years, 881 patients with spinal infections were treated either conservatively or surgically at our center, of whom 45 patients (45/881, 5.1%) had SIEA. The overall mean age was 69.6 ± 5.6 years of patients diagnosed with SIEA and all of them underwent posterior decompression via laminectomy. The mean Charlson Comorbidity Index (CCI) was 6.9 ± 2.5, indicating a poor baseline reserve. Progressive neurological decline was observed in all patients (mean motor score, 88.6 ± 9.7). The in-hospital rate and 90-day mortality were 4.4% and 10%, respectively. Mortality was not surgery related. Most importantly, the patients’ motor deficits and blood infection parameters significantly improved after surgery. Risk factors for mortality were increased age, comorbidities as measured by CCI, and preoperative motor weakness (MS).

**Conclusions:**

Immediate surgical decompression via laminectomy, with antiseptic irrigation and drainage of the subdural space, followed by antibiotic therapy, appears to be the key to ensuring beneficial clinical outcomes to treatment of rare diseases such as SIEA.

## Introduction

Spinal infection (SI) is a serious medical condition that affects the vertebral body, intervertebral discs, and paraspinal tissue.^1,2^ The incidence of SI is increasing, particularly in developed countries, presumably because of the improvement and availability of imaging diagnostics, an ever-aging population suffering from chronic illness, an increase in spinal surgeries, and the widespread use of intravenous drugs.^1-3^ The mortality rate of SI ranges between 2% and 30%.

An extremely rare form of SI is spinal intradural extramedullary abscess (SIEA).^4^ These abscesses are diffusely located in the subdural space. In contrast to spinal epidural abscesses, intradural abscesses can occur anywhere along the spinal cord, with a higher prevalence in the thoracic and lumbar spine.^2,5^ They are mostly caused by hematogenous infection, by spread of infection from osteomyelitis,^4^ or iatrogenic inoculation.^6^ When neurological deficits are present, emergency surgical drainage followed by appropriate antimicrobial therapy for 4-6 weeks has been proposed as standard treatment.^2,3,5,6^

The exact incidence of SIEA remains unclear, but it is associated with high morbidity and mortality rates, of up to 45%.^7-9^ However, evidence has predominantly been derived from case reports, and a systematic analysis is lacking. To date, only 63 cases have been reported^2,10^ and the clinical course and optimal management of this condition remain elusive.

Owing to the lack of robust evidence on this topic, we aimed to provide a systematic description of the clinical course of SIEAs in a large cohort with acute onset of neurological illness, to assess morbidity and mortality rates, and to determine potential risk factors for mortality associated with this condition.

## Methods

### Study Design, and Inclusion and Exclusion Criteria

**Figure 1.**
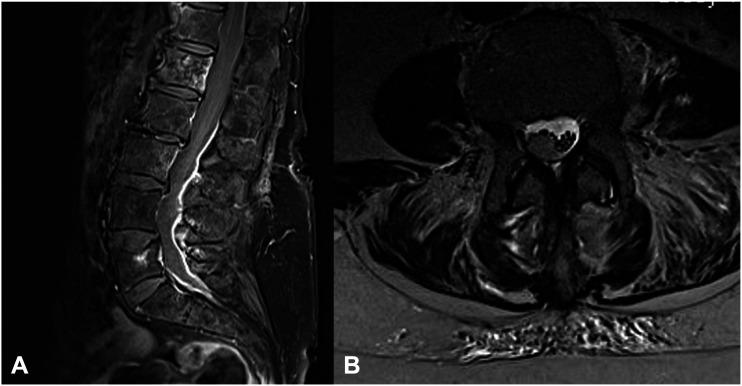
Postcontrast sagittal (A) (T1 gadolinium sequence) and T2-weighted axial (B) magnetic resonance imaging demonstrating pronounced meningeal enhancement and clear intramedullary compression with ring enhancement collection extending from L2-S1 in a65-year-old male patient presenting with lumbar pain and progressive lower extremity weakness.

Clinical and imaging data were retrospectively collected from our institution’s database between September 2005 and December 2020. Over this period, 881 patients with spinal infections were treated at our center, either conservatively or surgically- This study was approved by our institutional ethics committee (approval no. 880/2021) and was conducted in accordance with the Declaration of Helsinki. The requirement for obtaining informed consent was waived because of the retrospective nature of the study.Patients with SIEA anywhere along the spinal cord (cervical, thoracic, or lumbar) with no radiological signs of vertebral osteomyelitis were enrolled consecutively. The diagnosis was based on magnetic resonance imaging (MRI). Diffusion-weighted imaging was used to help differentiate the abscess from tumors, as previously recommended (Figure 1).^11^ Spine stability was examined using computed tomography (CT). The exclusion criteria were as follows: age < 18 years, concurrent intracranial pathology, spinal instability, and unavailability of the requisite data. Exclusion criteria related to spinal instability, based on CT or and MRI findings, were bony deconstruction resulting in kyphosis or subluxation of the vertebral column, any signs of vertebral osteomyelitis as determined on MRI and CT imaging by two independent spine surgeons with at least 15 years of experience (BI, KK), bone necrosis, and complete loss of disc height. Concerning the surgical technique, surgical exposure was achieved via laminectomy and a midline incision of dura. Neural elements were found to be enlarged with an altered appearance and an intradural extramedullary empyema could be excised in all of the cases. All cases were examined for radiological signs of spondylodiscitis by two experienced independent spine surgeons (BI, KK) Only three cases presented with mild signs of infection of the disc, as delineated on contrast-enhanced MRI imaging.

### Patient Characteristics

Data regarding patient demographics, comorbidities, American Society of Anesthesiologists scores, surgical duration, number of treated spinal levels, perioperative and postoperative complications, hospital length of stay, intensive care unit (ICU) stay, readmission, reoperation, and mortality were retrieved from the patients’ electronic records. Preoperative comorbidities were assessed using the age-adjusted Charlson Comorbidity Index (CCI). The CCI was calculated for each patient and was classified as no comorbidity, minimal comorbidity, moderate comorbidity, or severe comorbidity (CCI = 0, 1-2, 3-5, or > 5, respectively).^12,13^ The pre-treatment neurological condition was assessed using the motor score (MS) of the American Spinal Injury Association impairment grading system (MS = 0, no muscle strength; MS = 100, healthy). Posttreatment MS data were obtained from the last documented clinical encounter. In the current context, the ASIA motor score is a tool used by spine specialists to determine the extent of neurological disability.^14^ The ASIA motor score has been already validated in previous studies and provides reliable information for the evaluation of patients motor deficits.^15^ All patients presented with acute neurological decline and underwent posterior decompression via laminectomy within the first 24 h of admission. The attending spinal surgeon made the final decision to open the intradural space in all 45 cases. A primary dural closure was followed. Surgical decision-making was guided by presenting neurological status (i.e., MS), concomitant underlying pathologies, extent of pathology, disease prognosis, and the discretion of an experienced treatment team of neurosurgeons, neuroradiologists, and anesthesiologists.

Routine clinical and radiological follow-up examinations were performed before discharge and at 3 months postoperatively. The mean follow-up period was 33.4 months ± 12.1 months postoperatively. MRI was solely performed for clinical suspicion of abscess recurrence. In line with our institutional treatment protocols, blood samples or intraoperative cultures were collected prior to administration of IV antibiotics. Thereafter, IV antibiotics were initiated immediately. After identifying the bacterial specimens, the choice of IV antibiotics was adapted to reflect the antibiogram results. A lumbar puncture was not performed since it was not considered necessary for the therapeutic course. According to our institutional guidelines, vancomycin and meropenem were administered intravenously until the identification of the pathologic specimen, as previously suggested.^16,17^”

### Statistical Analysis

Categorical variables are presented as numbers and percentages. Continuous variables are presented as mean ± standard deviation. Normality of data distribution was assessed using the Shapiro–Wilk test. Baseline characteristics, duration of surgery, number of treated spinal levels, complications, length of hospital stay (LOS), ICU stay, readmission, reoperation, and mortality were compared group-wise using independent *t-*tests for continuous variables and chi-squared tests for categorical variables. The Wilcoxon rank test was used to evaluate changes in the C-reactive protein (CRP) level, leukocyte count, and neurological status (i.e., MS) of each group at discharge. In the second stage, binary logistic regression analysis was performed to identify potential risk factors for mortality. Statistical significance was set at a *P* value < .05.

## Results

### Demographic Data and Baseline Characteristics

Over a period of 15 years, 45 patients with a mean age of 69.6 ± 5.6 years, who were diagnosed with SIEA and who underwent posterior decompression via laminectomy and durotomy, were enrolled in this study. Over half of the patients were men (n = 27, 60%). The mean CCI was 6.9 ± 2.5, indicating a poor baseline reserve. Arterial hypertension, coronary heart disease, chronic obstructive pulmonary disease, renal failure, and diabetes mellitus were the most prevalent comorbidities (n = 35, 77.8%; n = 35, 77.8%; n = 34, 75.6%; n = 33, 73.3%; n = 18, 40%, respectively). The lumbar spine was the region most commonly affected (n = 19; 42.2%). Blood infection parameters were relatively high, with average CRP levels of 175.7 ± 12.5 mg/L and leucocytes 15.3 ± 6.9 cells/L. Progressive neurological progress was observed in all patients, with a mean MS of 88.6 ± 9.7, indicating the presence of a new motor deficit. A detailed breakdown of the patient characteristics is presented in Table 1.

**Table 1.** Baseline patient characteristics.

	Decompression N^a^ = 45
Age, y (mean, SD^b^)	69.6 (5.6)
Sex (n^c^, %)	
Male	27 (60.0)
Female	18 (40.0)
BMI, kg/m2 (mean, SD)	26.0 (3.4)
Comorbidities	
Age-adjusted CCI score (mean, SD)	6.9 (2.5)
Arterial hypertension (n, %)	35 (77.8)
Myocardial infarction (n, %)	6 (13.3)
Coronary heart disease (n, %)	35 (77.8)
Atrial fibrillation (n, %)	3 (6.7)
Heart failure (n, %)	4 (8.9)
COPD (n, %)	34 (75.6)
Diabetes mellitus Type II (n, %)	18 (40.0)
Renal failure (n, %)	33 (73.3)
Liver disease (n, %)	3 (6.7)
Gastrointestinal ulcer (n, %)	3 (6.7)
TIA/stroke (n, %)	1 (2.2)
Malignancy (n, %)	3 (6.7)
Dementia (n, %)	1 (2.2)
Previous spinal surgery (n, %)	
ASA class (n, %)	
I	2 (4.4)
II	11 (24.4)
III	26 (57.8)
IV	6 (13.3)
Localization of intradural abscess, (n, %)	
Cervical	7 (15.7)
Cervicothoracic	1 (2.2)
Thoracic	7 (15.7)
Thoracolumbar	1 (2.2)
Lumbar	19 (42.2)
Lumbosacral	8 (17.8)
CRP level, mg/L (mean, SD)	175.7 (12.5)
Leukocytes, count/L (mean, SD)	15.3 (6.9)
Preoperative MS score (mean, SD)	88.6 (9.7)

^a^group size.

^b^standard deviation.

^c^number of patients.

BMI, body mass index; CCI, Charlson Comorbidity Index; COPD, chronic obstructive pulmonary disease; TIA, transient ischemic attack; ASA, American Society of Anesthesiologists; CRP, C-reactive protein; MS, motor score of the American Spinal Injury Association grading system.

### Surgical Characteristics

As shown in Table 2, the mean surgical duration was 151.3 ± 113.8 min, with a mean blood loss of 433.3 ± 36.1 ml. The mean number of decompressed levels was 1.5 ± .7. The mean ICU stay was 27.3 ± 12.3 days, while hospital stays lasted 10.8 ± 14.3 days, on average. During hospitalization, two patients (4.4%) died because of severe respiratory decline caused by septic pneumonia. Both patients were aged 82 years and presented with a poor baseline reserve. In particular, both patients suffered from cardiovascular diseases such as arterial hypertonia, myocardial infarction, and atrial fibrillation, chronic end-stage renal failure, COPD, and type 2 diabetes mellitus. Besides these underlying diseases, a weakness in both lower extremities occurred in less than 24 hours (MS score 75). Considering the patients’ clinical and neurological status, the age-adjusted CCI was 13 points. After surgical decompression and evacuation of SIEA, both patients were transferred to the ICU. Although both patients received antibiotics for the SIEA, pulmonary problems requiring oxygen therapy developed. An X-ray scan of the thorax revealed pneumonia. Regardless of the increase in antibiotics administration, the infection levels in blood increased tremendously and both patients presented with clinically and laboratory confirmed sepsis. Unreassuringly, both patients died of rapid progression of pneumonia with renal and hepatic failure.The 90-day mortality was 10% and the cause of death was not associated with spinal pathology. No patient was readmitted, and no further surgeries due to secondary instability or abscess recurrence were required during the follow-up period (mean follow-up, 33.4 ± 12.1 months). Most importantly, the patients’ motor deficits and blood infection parameters improved significantly after surgery, as displayed in Table 3. Secondary instability did not occur in any case, as evaluated using X-ray and MRI at a mean follow-up of 33.6 months. *Staphylococcus aureus* was detected in almost the entire cohort (n = 36, 80.0%). The next most frequent pathogens were *Escherichia coli* and *Pseudomonas aeruginosa* (n = 3, 6.7%, respectively), followed by *Enterococcus faecalis* (n = 2, 4.4%). No pathogens were identified in one patient (n = 1, 2.2%).

**Table 2.** Comparison of surgical characteristics and clinical course.

	Decompression N^a^ = 45
Surgical duration, min	151.3 (113.8)
No. of levels decompressed/fused	1.8 (.7)
Hospital stay, days	27.3 (12.3)
ICU stay, days	10.8 (14.3)
Mortality	
In-hospital (n^b^, %)	2 (4.4)
90-day (n, %)	7 (15.6)
Post CRP	81.3 (37.3)
Post leukocytes	10.5 (3.7)
Post MS	92.3 (9.2)

Except where otherwise indicated, the quantities are presented as the mean (standard deviation, SD). *Significant difference; Post, after surgery; Delta, difference between pre- and post-surgical values. ICU, intensive care unit; CRP, C-reactive protein; MS, motor score of the American Spinal Injury Association grading system.

^a^group size.

^b^number of patients.

**Table 3.** Comparison at baseline (before surgery) and discharge.

	Decompression Baseline N^a^ = 45	Decompression Discharge N = 45	*P*-value
CRP	175.7 (12.5)	81.3 (37.3)	**<.001**
Leukocytes	15.3 (6.9)	10.5 (3.7)	**<.001**
MS	88.6 (9.7)	92.3 (9.2)	**<.001**

All data are presented as mean (SD). *significant difference. CRP, C-reactive protein; MS, motor score of the American Spinal Injury Association grading system.

^a^group size.

### Complications and Risk Factors for Mortality

The most prevalent complications were pleural effusion and deep wound infection (n = 9, 20%). Revision surgery was performed in three patients because of deep wound infection with cerebrospinal fluid (CSF) outflow. All nine patients with postoperative pleural effusion were older than 65 years of age, and had a poor baseline history such as congestive heart failure, elevated levels of proBNP, and COPD.Detailed data regarding the postoperative complications are presented in Table 4. In the second-stage analysis, potential risk factors for mortality were investigated. Interestingly, increased age and comorbidities, as measured by CCI, and preoperative motor weakness, as reflected by the MS, were associated with mortality, whereas the extent or duration of surgery, hospital stay, and occurrence of complications were not (Table 5).

**Table 4.** Occurrence of adverse events.

	Decompression N^a^ = 45
Deep wound infection	9 (20.0)
Acute respiratory failure	1 (2.2)
Acute heart failure	4 (9.1)
Acute renal failure	3 (7.8)
Septic shock	2 (4.4)
Pneumonia	7 (15.6)
Pleural effusion	9 (20.0)
Ileus	1 (2.2)
Urinary tract infection	5 (11.1)

All data are presented as number of patients (%). *significant difference.

^a^group size.

**Table 5.** Risk factors associated with mortality.

Risk Factor	OR (95% CI)	*P*-value
Age	1.4 (1.1-3.4)	**.004**
Age-adjusted CCI score	1.7 (1.1-4.9)	**.032**
Preoperative MS	1.3 (1.1-1.8)	**.004**
Duration of surgery	1.1 (1.0-1.3)	.986
Number of levels decompressed	.3 (.1-.9)	.089
Length of ICU stay	.3 (.2-1.1)	.201
Length of hospital stay	1.1 (.8-1.6)	.893
Complications	1.5 (1.0-2.7)	.065

CCI, Charlson Comorbidity Index; CI, confidence interval; ICU, intensive care unit; MS, motor score of the American Spinal Injury Association grading system; OR, odds ratio; PTT, partial thromboplastin time.

## Discussion

SIEA is a rare and unpredictable type of spinal infection. To date, the exact incidence and natural course of the disease has remained unknown.^8,9,18^ It is considered a neurosurgical emergency, requiring prompt diagnosis and immediate drainage—within 24 h—of the intradural abscess, aiming to preserve the patients’ neurological condition and avoid disease progression.^9,11^ Notwithstanding, the current understanding of the pathophysiology, diagnosis, and management is based on anecdotal evidence owing to the rarity of the disease.^5,9,18,19^

### Summary of Findings

To the best of our knowledge, no previous study has systematically examined such a large cohort of patients with SIEA who underwent surgery. We assessed morbidity and mortality rates and determined potential risk factors for mortality in patients undergoing posterior surgical decompression and intradural drainage of the abscess within the first 24 h after the first indication of acute onset of neurological deterioration. We found high comorbidity rates, as defined by the age-adjusted CCI, which indicated poor baseline reserve of the enrolled patients. The most prevalent location of SIEA was the lumbar spine (42%). Notably, all patients presented with progressive motor weakness and high levels of infection, as determined by laboratory examinations. The in-hospital and 90-day mortality were relatively high at 4.4% and 15.6%, respectively. Post-surgery, the levels of infection and patients’ neurological condition improved significantly, with a mean MS of 92.3. Interestingly, significant factors associated with mortality were older age, high comorbidity rates, and high-grade motor deficits. In a mean follow up period of 33.4 months, no further surgery due to secondary instability or abscess recurrence was necessary.

### Literature Review

Patients with poor clinical vignette and increasing age are at a higher risk of spinal infections.^2,3,20,21^ Comorbidities, such as obesity, diabetes mellitus, renal failure, intravenous drug abuse, chronic infection, immunodeficiency, and previous spinal surgery, are considered pivotal factors for SI^2^ For instance, a large retrospective study based on claims data of 228,044 patients with spondylodiscitis reported that increased age and higher CCI were significant risk factors for mortality. Notably, congestive heart failure, cerebrovascular disease, liver disease, and renal disease were found to be significant predictors of mortality.^22^ Because SIEA is a major component of SI, one might assume that the above-mentioned compounding factors might also be pertinent to this rare but devastating illness. Lange et al. conducted a retrospective study of 16 cases of spinal abscess with a mean age of 57.9 years (range 32-73 years) and confirmed that underlying illnesses, such as diabetes mellitus, liver disease, renal failure, or immunodeficiency, were critical factors associated with patients’ clinical outcome after surgery.^9^ However, it is important to highlight that, owing to the imaging quality of the MRIs from the early 90 s, it was not possible to distinguish between extra- and intra-dural abscesses. However, intraoperative findings revealed that only two of the 16 cases evaluated by Lange et al. had an intradural extramedullary abscess. A detailed description of the clinical course of these two cases was not provided. Similar to the findings of Lange et al., Agarwal et al., in a case report of SIEA, advocated that previous immunodeficiency due to chronic infection, such as HIV or hepatitis C virus infection, might be inciting events that could cause even a young 37-year-old patient to be susceptible to SI, and even to develop an intradural extramedullary mass.^5^ In another case report on SIEA, diabetes mellitus was the underlying condition of a 55-year-old male patient, which presumably contributed to the development of SIEA.^7^

Nevertheless, all existing literature involved case reports. Thus, it was difficult to draw conclusions, and particularly to determine the potential impact of comorbidities on patient outcomes. The findings of the present study, which was a systematic analysis, affirmed the notion of a potential relationship between underlying diseases and SIEA. We showed that the patients were weakened by their poor baseline history with a CCI of almost 7. Interestingly, in our cohort, we did not have any patients with a history of intravenous drug abuse or HIV disease. This phenomenon might be related to the mainly older individuals in our cohort, in which only 15.6% were 50 years or younger. The most prevalent comorbidities were cardiovascular disease, renal failure, and diabetes mellitus. It is important to highlight that both increased numbers of older patients and higher rates of comorbidities were significant risk factors for mortality. Therefore, it appears that, in the case of SIEA, the same patient characteristics as in all other SIs should be thoroughly monitored, and a frank discussion should be had with patients and their relatives before establishing a treatment plan, since these patients are inevitably at higher risk of mortality.

According to the findings of the present study, nine patients aged 65 years and older developed pleural effusion. Herein, it is important to highlight that these patients had congestive heart failure and COPD as underlying diseases; hence, they were susceptible to postoperative respiratory complications. Potential and widely-accepted explanations are increased capillary pressure, as seen in congestive heart failure; 2) increased capillary permeability, which occurs in inflammatory conditions such as pancreatitis, peritonitis, and pneumonia; 3) conditions that lead to a decreased colloidal osmotic pressure, such as hypoalbuminemia; 4) increased negative intrapleural pressure, as seen in atelectasis. These phenomena presumably have occurred in this subset of patients, who after surgery presumably decompensated.^23^ The development of pleural effusion should be underestimated since it is associated with increased mortality rates ranging between 25% and 57%.^24^ Therefore, older patients with poor baseline reserve should be closely monitored after a surgical procedure, even when placed in the ICU, to prevent such complications that might lead to death

The collection of pus in the intradural space causes space-occupying lesions, resulting in pain, radicular symptoms, or motor weakness. According to Bartels et al., symptoms of SIEA can be classified into three different stages, with fever and pain being the first, neurological decline being the second, and paralysis and complete sensory loss below the level of the lesion being the last.^7^ Notably, the progression of symptoms is not predictable; thus, prompt diagnosis and a swift start of therapy are warranted. In the present series, all patients presented with fever, substantially elevated blood infection parameters, and, most importantly, progressive neurological deterioration. In this context, an MRI of the spinal cord was performed, revealing the presence of the intradural abscess. Since neurological deficits lasting longer than 24-36 h have potentially poor prognosis, urgent surgical decompression and abscess drainage appear to be the optimal treatment choice. Nevertheless, since our cohort consisted of patients with poor baseline reserve, a thorough discussion with anesthesiologists was necessary to determine potential intra- and perioperative complications. Surgery was chosen for all patients, and a significant improvement of both blood infection values as well as of the neurological condition was noted already at discharge, with the simultaneous administration of intravenous antibiotics.

However, to date, the guidelines concerning the optimal therapy for such a devastating illness are anecdotal and are based on the physician’s experience. In a case report of a 42-year-old woman diagnosed with SIEA at the L3–L4 level, with progressive motor weakness of the lower limbs, Thomé et al. reported significant mitigation of the deficits after surgery with a complete diminution of the lesion on MRI and clinical resolution at the 12-month follow-up.^6^ In conjunction with these findings, in a similar case of a middle-aged male patient suffering from SIEA at the Th12 level, with paraparesis of the lower extremities, immediate surgical decompression and pus evacuation led to significant improvement of the neurological status and diminished the patient’s severe low back pain.^25^ Bartel et al. reviewed 45 patients treated for SIEA and stated that surgical decompression and abscess evacuation might be the key tools for the recovery of patients’ neurological condition, while conservative management contributed not only to poor clinical outcomes, but was also associated with substantially high mortality rates of almost 80%.^7^ Although a clear consensus is still lacking, it appears that surgical decompression with concomitant antibiotic treatment might be a tool for regaining the well-being of such patients.

SIEA is a very rare disease and its exact incidence remains unknown, with fewer than 65 cases reported in the literature to date.^7,26,27^ Bartel et al., in reviewing 45 cases of SIEA, reported that mortality occurred in six of 39 patients (15.4%) who underwent surgery, without specifying the exact cause of death.^7^ In contrast, 20% of the conservatively treated patients died, which is a much higher rate than that reported for the surgical cohort.^7^ In the present study, the in-hospital mortality was relatively low, at 4.4%, while the 90-day mortality rate increased steeply to 15.6%, and the 3-year mortality remained stable at 17.8%; these findings are similar to those of Bartels et al.^7^ The much lower in-hospital mortality rate might be attributable to the postoperative care of the patients. All patients in our department were postoperatively transferred to the ICU for monitoring and better management of any potential complications owing to the complexity of the disease itself, as well as their history of comorbidities. It should be noted that ICU utilization is associated with high costs and a high demand for resources. Such care is more feasible in Western countries, while hospitals in rural areas lack such facilities, and thus the mortality rates of octogenarians might be higher in such areas.^28^ Importantly, the reported deaths were not surgery-related, but were because of unsolicited events associated with the patients’ poor baseline reserve. During hospitalization two patients died because of respiratory failure caused by septic pneumonia, while in the follow-up period three patients died owing to heart failure—two because of pulmonary embolism and three owing to ischemic stroke. No further surgeries due to secondary instability were required, and no disease recurrence was observed after surgical management. According to the findings of the present study, two older patients aged>80 years died of progressive septic pneumonia. In our regression analysis, age, neurological status, and rates of comorbidities were significant predictors of patient mortality, whereas surgical procedure and duration of surgery were not. Both deceased patients presented with a significantly poor baseline reserve before surgery, which conferred on them higher risks of postoperative mortality. Considering these points, one might argue that in octogenarians, spinal surgery should be thoroughly discussed with the patients and their families since postoperative decompensation might presumably occur; hence, such patients not only susceptible to complications but have a risk of death. Lenga et al. in their retrospective study exclusively on octogenarians with spinal epidural abscess in the thoracic and lumbar spine stated that surgery leads to improvements in infections levels and neurological status, although the potential complications after surgery, such as pneumonia or septic shock, should not be underestimated in this unique subset of patients with multiple needs.^29^ Furthermore, findings from our study group showed that octogenarians with a spinal infection have a significantly higher risk of mortality and postoperative complications compared to their younger counterparts.^30^ Unreassuringly, data on SIEA in older patients is lacking and current knowledge is based solely on case reports.^6,7^ Notwithstanding, as regards octogenarians with an acute neurological decline, which is in due to spinal infection in this case, a devastating course should be expected. This is why a clear discussion with patients and their families should be conducted before surgery, and particularly, the patient’s will should be meticulously interpreted.

To shed further light on potential factors associated with mortality, we performed a logistic regression analysis and showed that increased age, higher rates of comorbidities, and worse pre-existing motor deficits were critical factors related to mortality. Considering these points, it seems that, on one hand, emergency surgical decompression and evacuation might be adapted as the “state of the art” treatment, while on the other hand, patients’ frailty and age should be considered, and the treatment strategy should meticulously be discussed with the different disciplines in order to choose a strategy where the benefits outweigh the shortcomings.

### Strengths and Limitations

The main strength of the current study is that it systematically examined a large cohort of 45 patients diagnosed with this rare condition, and assessed the clinical course and outcomes of SIEA, which has not been reported previously. Since the disease is rare and data comes mainly from case reports,^4,6,7,18^ a standardized diagnostic and treatment algorithm is still missing. For instance, Konovalov et al. described a case of SIEA that was initially thought to be an intradural tumor, thereby delaying the initiation of antibiotic therapy.^18^ In presence of new acute neurological signs, an MRI or CT of the spine is typically performed. However, the absence of contrast-enhanced MRI or CT sequences will mislead the diagnosis; hence a significant delay in the therapeutic procedure might occur, resulting in worse outcomes. In the present study, a contrast-enhanced MRI of the spine revealed the pathology. The suspicion on spinal infection was also attributable to the acute neurological deterioration of the patients. However, in the absence of fever or other clinical signs of infection, the initial diagnosis might be false. Therefore, we believe that in case of acute neurological deficits, spinal infection and even the rare condition SIEA should be considered; hence, a contrast-enhanced MRI should be performed as part of the routine diagnostic work-up. Afterward, emergent surgery with the intercurrent administration of intravenous antibiotics might be appropriate for such devastating condition. Prospective studies are warranted to substantiate our findings and to develop guidelines for the optimal therapy for this disease under consideration of older patients with the highest risks of morbidity and mortality, with respect to the poor baseline history.

Our institution is one of the largest neurosurgical and spine centers in Germany, performing over 800 surgeries for spinal pathologies each year. It is important to emphasize that we analyzed cases over a 15-year-period and included 45 cases in our analysis. Smaller hospitals in a radius of more than 50 km refer patients to our institution for therapy owing to the limited resources or lack of expertise at their hospitals. Similar to the number of cases in our study, Bartel et al. reviewed approximately 60 cases over a 10-year period.^7^ Furthermore, high-quality imaging allows us to diagnose such pathologies which in other centers may overlook due to limited resources as well as the false diagnosis of SIEA. Despite these points, the rarity of the diseases makes it less appealing to investigate since a multicenter approach or large single center institutions are warranted to acquire a substantial number of patients. As a result, this devastating illness has thus far only played a marginal role in clinical studies or has been a by-product of reports on spinal infections. Therefore, the last systematic reviews on the topic was published in the early 2000 or even the 90s.^7,19^

However, this study had some limitations. First, it may be argued that a relatively small cohort of patients was investigated. However, since previous data were derived only from individual case reports, we feel that our findings provide a real-world picture of the disease and a basis for developing guidelines concerning its epidemiology and treatment. Second, selection bias may have been present because of the retrospective study design. Third, the minimum follow-up period of 12 months was relatively short. By gathering longer-term data, other relevant findings that were not captured in the current study might have been revealed. Due to the retrospective study design of the study, the MS by the ASIA was employed to determine the motor status of patients. A detailed description of patients’ neurological condition might be useful to gain more insight of the pathology. However, since this score has routinely been used in previous studies and its robustness has been validated, we believe that its use provided substantial information on patients’ clinical status and allowed the investigation of potential associations between neurological status and patient outcomes. Functional outcomes could not be sufficiently reconstructed using patients’ medical records; hence, they were omitted from this analysis. However, we believe that the impact of surgery could be adequately evaluated using the ASIA grading system.Larger studies are warranted to elucidate the mechanisms underlying the pathogenesis of SIEA further and to establish morbidity and mortality rates.

## Conclusions

SIEA is an extremely rare pathology encountered in spinal surgery. Notwithstanding, surgeons are confronted with its therapeutic management, and the existing knowledge is based on their experience. The findings of the present study showed that prompt decompression (laminectomy) with antiseptic irrigation and drainage of the subdural space, followed by antibiotic therapy, might be the key tool for treating this rare disease. To achieve an adequate eradication of SIEA, the extent of surgery required decompression and then abscess evacuation should be at least 1.8 levels. However, increased age, preoperative neurological deficits, and comorbidity rates should be considered carefully, as these factors are strongly associated with disease prognosis and patient mortality.
